# The role of prophylactic central compartment lymph node dissection in elderly patients with differentiated thyroid cancer: a multicentric study

**DOI:** 10.1186/s12893-018-0433-0

**Published:** 2019-04-24

**Authors:** Claudio Gambardella, Renato Patrone, Francesco Di Capua, Chiara Offi, Claudio Mauriello, Guglielmo Clarizia, Claudia Andretta, Andrea Polistena, Alessandro Sanguinetti, Pietrogiorgio Calò, Giovanni Docimo, Nicola Avenia, Giovanni Conzo

**Affiliations:** 1 Division of General and Oncologic Surgery, Department of Traslational Medical Sciences, University of Campania “Luigi Vanvitelli”, School of Medicine, Via Sergio Pansini 5, 80131 Naples, Italy; 2 Endocrine Surgery Unit, University of Perugia, Piazza dell’Università, 06123 Perugia, Italy; 3 Department of Surgical Sciences, University of Cagliari, Cagliari, Italy

**Keywords:** Total thyroidectomy, Differentiated thyroid cancer, Prophylactic central neck dissection, Elderly patients

## Abstract

**Background:**

Prophylactic central neck lymph-nodes dissection is still a topic of major debate in Literature. There is a lack of randomized controlled trials proving advantages in its application in terms of overall survival and local recurrence. Due to the recent rapid increase of elderly population, differentiated tumor carcinoma diagnosis increased in patients over 65 years old. The aim of this study was to compare recurrence rate, complications rate and histological features of tumors in elderly population.

**Methods:**

A retrospective study was carried out collecting data from 371 patients with differentiated thyroid cancer without clinical evidence of lymph-nodes involvement in three Italian referral centers from 2005 to 2015. All patients were aged ≥ 65 years and were divided in two groups based on the performed surgery (total thyroidectomy alone or associated with central lymph-nodes dissection). Moreover, patients were stratified according to the age between 65 and 74 years old and over 75 years old.

**Results:**

Total thyroidectomy alone was performed in 184 patients (group A) and total thyroidectomy with prophylactic central neck dissection was performed in 187 cases (group B). There was a statistically significant difference in complications between the groups in terms of neck hematoma (0.5% group A vs 3.7% group B), temporary hypoparathyroidism (11.4% group A vs 21.4% group B), and temporary unilateral recurrent nerve injury (1.5% group A vs 6.4% group B). Lymph nodes recurrence rate was 9.2% in group A and 8.5% in group B, with no statistically significant difference. There was a statistically significant difference in patients over 75 years old in terms of temporary hypoparathyroidism (24% group A vs 11% group B), permanent hypoparathyroidism (2,7% group A vs 0,3% group B) and recurrent nerve injury (9,5% group A vs 2% group B).

**Conclusions:**

The role of prophylactic central neck dissection is still controversial, especially in elderly patients, and an aggressive surgical approach should be carefully evaluated. The Authors reported a similar low recurrence rate between total thyroidectomy and total thyroidectomy associated with prophylactic central neck dissection, with increased postoperative complications in the lymphadenectomy group and in patients over 75 years old, advocating a tailored surgical approach in elderly population.

## Background

Differentiated thyroid cancer (DTC) is the most common tumor among endocrine malignant pathology with an incidence that has dramatically increased in the last few decades (almost 310% from 1950 to 2004). In Italy, there were almost 15,300 new cases of thyroid cancer during 2016, representing 4% of all malignant neoplasm with a 1:4 male/female ratio. Despite the increased incidence, mortality did not raise accordingly. Thyroid cancer has a peak of incidence between 45 and 55 years with 5:10000 cases per year. Below 45 years old and above 55 years old, the incidence decreases reaching 1:10000 cases per year in over 75 years old population [1].

DTC has generally an indolent clinical course, an excellent prognosis and is associated with a low mortality rate witch lead to a “crafty” approach to this pathology. In fact, well-differentiated (papillary and follicular) thyroid cancer had an expected 20-year survival of 90% or greater, exclusive of other causes of mortality [2]. Unfortunately, the long-term recurrence rate is not negligible (15–30%), even more if we consider the regional lymph node micro metastases, which are involved in up to 80% cases, defining the high recurrence rate as the main obstacle for clinical node-negative (cN0) patients [3, 4]. Prophylactic central neck dissection (pCND) is defined as the complete excision of level VI and VII lymph nodes (based on the recognized anatomic continuity from the neck and superior mediastinum) in patients with no evidence of nodal involvement and may be performed safely in referral centers [5]. Several surgeons suggested that pCND associated with total thyroidectomy (TT) should be performed in cN0 patients to avoid the locoregional recurrence and provide pathological evidence for the adjuvant radioiodine (RAI) treatment [6, 7]. Certainly, tumor histotype influences the survival and recurrence rate, representing the most significant prognostic factor. According to ESMO (European Society of Medical Oncology), BTA (British Thyroid Association), ATA (American Thyroid Association), NCCN (National Comprehensive Cancer Network), therapeutic central neck dissection should be performed in case of a clinical or ultrasonographic evidence of lymph nodes metastases in central compartment [8–11]. However, the role of pCND remains controversial in patients without clinically evident lymph nodes metastasis. Due to the recent rapid increase in the life expectancy of the general population, the elderly population has growth by 90% over the last 30 years [12]. Elderly patients are conventionally defined as those with an age of 65 years or over [13]. Nevertheless, this definition is not worldwide accepted, thus several Authors have proposed a different cut-off at the age of 75 years old (very old patient) [2]. Recent papers described a dissimilar incidence of lymph node recurrence after DTC in aged people compared to young ones without reaching definitive conclusions [2, 12–14]. The implications for recurrent disease are very different among elderly and younger population. Moreover, assessing the rate of recurrence of DTC and the impact of thyroid cancer recurrence on mortality is made more challenging because recurrence and mortality are competing risks. The aim of this study was to evaluate the incidence, the risk factors and the recurrence of central compartment lymph nodes in elderly patients (age ≥ 65 years) with cN0 PTMC.

## Methods

### Study design

Data of elderly patients’ undergone total thyroidectomy (TT) between January 2005 and December 2015 in three referral centers for endocrine surgery were retrospectively assessed. 371 DTC patients with clinically negative lymph nodes who underwent surgery with curative intent, were enrolled in the study. Inclusion criteria were age ≥ 65 years with a fine needle cytology (FNC) proven DTC, tumor with cN0 and ≥ cT1, no history of head and neck surgery or radiation and no history of other tumors. All patients had a documentation of a normal vocal cord mobility by preoperative laryngoscopy. A preoperative neck ultrasound was required to assess status of thyroid, central and lateral cervical compartments. FT3, FT4, TSH, Tg and anti-Tg antibody levels were also evaluated. Exclusion criteria were the presence of lymph node metastases in the central or in the lateral compartment discovered during preoperative investigations or during surgery. Other exclusion criteria were primary hyperparathyroidism, previous cervical surgery or radioactive iodine therapy. The following information were retrospectively collected from the medical records of the patients: gender, age, tumor size, bilaterality, multifocality, lymph nodes metastasis, capsular invasion, extra thyroidal invasion, cTNM and pTNM, postoperative complications.

### Patients

The patients were divided into two groups: the patients of group A underwent TT and the patients of group B underwent TT with prophylactic lymphadenectomy of the bilateral central neck compartment. The group A procedures were performed by one surgery unit (Napoli University) while group B procedure were performed by two surgery units (Perugia and Cagliari University). All the procedures in each single center were performed by the same experienced team with an average of more than 200 thyroidectomies per year (with a 20–25% rate of cancers). Moreover, in each group, patients were stratified according to the age between 65 and 74 years old and over 75 years old (very old people). Written informed consent was obtained for all patients before surgery.

### Surgical technique

Every TT was performed by experienced endocrine surgeons and with standardized surgical technique. In some cases, an ultrasound scalpel (Harmonic Ace; Ethicon Endosurgery, Blue Ash, Cincinnati, OH) was used and hemostasis was completed with the use of Floseal Hemostatic Matrix (Baxter, Zurich, Switzerland). Recurrent laryngeal nerve was routinely bilaterally identified until its insertion into the larynx. In case of removal or injury of the parathyroid glands, the auto-implantation was carried out at the level of the stern-mastoid muscle. The central neck compartment lymph node dissection included prelaryngeal, pretracheal and paratracheal lymph nodes basin on the ipsilateral and contralateral side to the tumor. Drainage was used selectively in group B and it was not used in group A. Serum calcium level was evaluated in the first two postoperative days and after one week from surgery.

### Radioiodine ablation

After surgery, adjuvant radioactive ablation (RAI) was administered in case of tumor size > 1 cm, distant or locoregional extension, non-papillary histologic subtype, multifocal disease. Adequate levels of TSH (> 30 mU/mL) were obtained by ceasing for 4 weeks thyroid hormone therapy or using recombinant human thyrotropin (rhTSH; Thyrogen, Genzyme Corp). Whole body scans were performed after RAI therapy to evaluate disease persistence.

### Follow up

Tumor size, extension, lymph node metastases and adjacent or distant organs involvement were evaluated using the American Joint Committee on Cancer TNM Classification of Thyroid Cancer. Post-operative diagnosis of central neck lymph nodes recurrence was obtained during follow up (6 months after surgery during suppressive L-thyroxine treatment) using US-guided FNAC on suspicious lymph nodes (≥1 cm) in patients with high levels of serum Tg (> 1 ng/mL). Major perioperative and postoperative complications found were neck hematoma requiring reoperation, transient or permanent unilateral or bilateral recurrent nerve injury and transient or permanent hypoparathyroidism. Hypoparathyroidism was considered permanent if it lasted more than 6 months and required medical therapy with normal levels of parathyroid hormone serum levels. Paralysis of the recurrent laryngeal nerve was confirmed by laryngoscopy and it was considered permanent if it persisted for more than 6 months.

### Statistical analysis

All statistical data was obtained using SPSS 24 software. Data were compared with a chi-square test. Statistical significance was defined as *p* < 0.05 with a Confidence Interval (CI) at 95%.

## Results

Demographic data of the 371 DTC patients are reported in Table 1.

**Table 1:** Demographics and pathologic findings

	Group A	Group B	*p* Value
Patients	184	187	
Male/ Female ratio	46/138	53/134	*0.466*
Age 65–74	152	146	*0.272*
Age 75+	32	41	
Histologic Features:			
• Microcarcinoma %	29.8	31.5	*0.729*
• Unifocal %	84.2	86.1	*0.614*
• Multifocal %	15.7	13.9	*0.614*
pTNM stage %			
• I	91.8	88.2	*0.245*
• II	5.9	8.5	*0.339*
• III	2.1	3.2	*0.538*

TT was performed in 184 patients (49.5%; group A), and TT with pCND was performed in 187 cases (50.5%; group B). The two groups were similar in demographics, clinical and pathologic findings. The main histological feature was unifocal tumor (85% of cases) with a stage I disease (90% of cases). PCND identified unexpected positive lymph node metastases in 41 of 187 cases (21.9%). In 29 (15.5%) of these patients, tumors > 1 cm were found, whereas tumors < 1 cm were found in 12 (6.4%) cases (*p* < 0.05). In 33 of 371 patients (8.8%) (14/184 or 7.6% in group A and 19/187 or 10.1% in group B) parathyroid tissue was implanted in the sternomastoid muscles, and in 42/371 (11.3%) cases parathyroid tissue was identified in the final pathology analyses. There was a statistically significant difference in complications between Group A and Group B patients in terms of neck hematoma (0.5%vs 3.7%, *P* = 0.03), temporary hypoparathyroidism (11.4% vs 21.4%, *P* = 0.009), temporary unilateral recurrent nerve injury (1.5% vs 6.4%, *p* = 0.019) (Table 2).

**Table 2:** Postoperative Complications

	Group A (*n* = 184)	Group B (*n* = 187)	*p* Value
Neck hematoma %	0.5	3.7	0.033*
Hypoparathyroidism %			
• Temporary	11.4	21.4	0.0095*
• Permanent	0.5	2.1	*0.182*
Recurrent nerve injury %			
• Temporary Unilateral	1.5	6.4	0.0193*
• Permanent Unilateral	0	0.5	*0.320*
• Bilateral VCP	0	0	*0.571*

*Statistically significant

In 4 cases of 371 patients (1%, 1 in group A and 3 in group), patients underwent neck re-exploration for severe neck hematoma.

### Follow-up and oncologic outcomes

Lymph nodes recurrence rate was observed in 17 of 184 cases of the group A (9.2%, 10 central recurrence and 7 ipsilateral recurrence) and in 16 of 187 cases of the group B (8.5%, 8 central recurrence and 8 ipsilateral recurrence). The clinicopathological findings of these patients are reported in Table 3.

**Table 3:** Locoregional relapse: patient demographics and pathologic findings

	Group A	Group B	*p* Value
Central recurrence	10 (5.4%)	8 (4.2%)	*0.604*
Lateral recurrence	7 (3.8%)	8 (4.2%)	*0.816*
Mean Age (years)	70.2 ± 4.6	72.3 ± 5.8	*0.648*
Male/Female	7/17	8/15	*0.679*
Mean elapsed time (months)	33 ± 3	37 ± 6	*0.772*
Follicular Variant	3	2	*0.680*
Classic Variant	14	14	

The two groups exhibited similar incidences of central and ipsilateral compartment recurrence and elapsed time between the primary operation and the recurrence, with no statistically significant difference. In the 85% of cases, the histopathological variant was papillary thyroid carcinoma. On 371 patients, 198 (53%) completed 5 years follow up. None of the enrolled patients died due to DTC-related recurrence or distant metastasis during the follow up period. After surgery, 321 patients (86.5%) underwent RAI.

### Analysis of patient pathologic findings stratified by age

Pathologic findings and complications of patient stratified for different ages were compared and collected in Table 4.

**Table 4:** Recurrence rate and clinicopathological findings of patient stratified for different ages

	Age 65–74 (*n* = 298)	Age 75+ (*n* = 73)	*p* Value
Central neck recurrence	12 (4%)	6 (8.2%)	*0.135*
Lateral recurrence	11 (3.6%)	4 (5.4%)	*0.486*
Histologic Features:			
• Mean tumor size (mm)	17 ± 11	22 ± 5	*0.513*
• Microcarcinoma	98 (32.8%)	16 (21.9%)	*0.068*
• Unifocal	276 (92.6%)	62 (84.9%)	< 0,001*
• Multifocal	22 (7.3%)	11 (15%)	< 0,001*
pTNM stage			
• I	280 (93.9%)	54 (73.9%)	< 0.001*
• II	15 (5%)	12 (16.4%)	< 0.001*
• III	3 (1%)	7 (9.5%)	< 0.001*
Complications (All patients)			
• Temporary hypoparathyroidism	34 (11.4%)	18 (24.6%)	0.003*
• Permanent hypoparathyroidism	1 (0.33%)	2 (2.7%)	0.039*
• Recurrent nerve injury	6 (2%)	7 (9.5%)	0.001*
Complications (Group A - TT)			
• Temporary hypoparathyroidism	12/152 (7,8%)	6/32 (18,7%)	*0.060*
• Permanent hypoparathyroidism	0/152 (0%)	0/32 (0%)	*NA*
• Recurrent nerve injury	2/152 (1,3%)	3/32 (9,7%)	0.010*
Complications (Group B – TT+ PCLD)			
• Temporary hypoparathyroidism	22/146 (15%)	12/41 (29,2%)	*0.037*
• Permanent hypoparathyroidism	1/146 (0,6%)	2/41 (4,8%)	*0.059*
• Recurrent nerve injury	4/146 (2,7%)	4/41 (9,7%)	0.049*

*Statistically significant

Considering complications, multifocality and tumor stage, there was a statistically significant difference in patients over 75 years old in terms of temporary hypoparathyroidism (24% vs 11%; *p* = 0,003; CI = 95%), permanent hypoparathyroidism (2,7% vs 0,3%; *p* = 0.03; CI = 95%), recurrent nerve injury (9,5% vs 2%; *p* = 0,001; CI = 95%), tumor multifocality (15% vs 7%; *p* = 0.03; CI = 95), tumor stage II (16,4% vs 5%; p = 0,0008; CI = 99%), tumor stage III (9,5% vs 1%; p = 0,0003; CI = 99%). Conversely, there was a statistically significant higher incidence of tumor stage I in 65–74 years old group. There were no statistically significant differences in microcarcinoma rate and in tumor size, even if patients over 75 years old presented slightly larger tumors.

## Discussion

The treatment of DTC have changed drastically in last 10 years due to new evidence-based clinical data and constant guidelines revisions [10]. Nevertheless, there are still many controversial aspects matter of intense debate in Literature. PCND is one of the major discussed topic. In fact, while there is a common consensus about recommendation of lateral neck dissection in case of clinically involved lymph nodes, several surgeons, mainly in Eastern countries, perform routinely pCND associated with TT in all patients with DTC [15]. The most likely explanations are that ultrasonography (US) might undervalue smaller lymph nodes, and, in case of central lymph node recurrence, reoperation is associated with higher rate of severe complications such as recurrent laryngeal nerve injury [16]. According to ATA 2015 guidelines, there is a strong recommendation to not perform pCND for smaller DTC (T1-T2 cN0) while it should be considered (weak recommendation) in case of a laterocervical lymph node involvement (cN1b), of a T3-T4 tumor or in order to stage the neoplasm and plan a subsequent RAI ablation. In DTC, lymph node metastases rate ranges from 20 to 50% with a micrometastases (size < 2 mm) rate being as high as 90% with a subsequent locoregional recurrence rate of 15–30%, while central compartment recurrence rate, ranges between 5 and 20% in 5–10 years [6, 17–20]. While performing a pCND potentially treat the local disease decreasing the recurrence rate, it will not change the overall prognosis [21]. This is the main reason, along with the increased postoperative complications, which brought Authors to debate about usefulness of pCND in clinically negative lymphnodes patients [22]. Patients who underwent TT without pCND presented a low risk of locoregional recurrence and improved staging seems to be the only advantages of this approach [5]. Moreover, pCND might cause upstaging of the tumor and potential overtreatment by the administration of RAI ablation [5].

This hot topic become even more debated in case of elderly patients. In fact, in younger population where thyroid cancer is generally diagnosed, it would be rare for a patient not to outlive the recurrence. However, in a study population whose average age is over 70 years, this may not be the case because many elderly patients are not living long enough for recurrence to become a reality. Recurrence, then, in the elderly population becomes an indicator that a patient has survived longer [22].

DTC is the most common malignancy of the endocrine system, and usually, is highly treatable and curable. Tumor stage, extrathyroidal extension, stage of disease, sex and age have been advocated as potential risk factors influencing prognosis and oncologic outcome [22]. The prevalence of DTC grow consensually to the age, which was considered a major prognostic factor in several large studies [14, 23–25]. In Literature are reported several staging systems considering different cutoff age such as 45, 60, 65, 75 or 85 years old due to the lack of a precise definition of old age [12, 26, 27]. Aging was already described to be a risk factor for aggressive thyroid pathology associated with worst prognosis (ATA 2015) and different recent works described the role of age as a major independent prognostic factor in central compartment lymph node recurrence [28–31]. Very old patients have generally more aggressive clinicopathological features such as tumor size, extrathyroidal extension, T status and lymphnodes metastases, probably for the delayed diagnosis [2, 14]. Chereau et al. described a more aggressive tumor behavior in elderly patients with a higher recurrence rate of lymph node metastasis and a lower disease free survival compared to young patients [2]. In particular, the patients over 75 years old were affected by a 2-fold increased risk of recurrence compared with younger patients. Similar findings were reported by Niemann et al., who described a very aggressive pattern among patients with DTC aged under 25 years old and over 75 years old [32]. The former Authors supported a more aggressive surgical approach in DTC patients, suggesting to perform a lymph node dissection along with TT in all patients over 75 year old, especially if RAI ablation would supposed to be problematic and not administrated in this population [2]. In fact, postoperative RAI therapy is affected by several severe complications among elderly population, such as insomnia, osteoporosis and arrhythmias, and is generally administrated with a lower dose [33, 34]. Moreover, Biliotti et al. reported that the effectiveness of radioiodine therapy decreases in the elderly, postulating that uptake of 131I is age dependent, probably for to the diminished differentiation of follicular and papillary carcinomas in geriatric patients [35]. Several studies focusing on DTC elderly patients reported a longer overall survival and disease free survival in patients treated by an aggressive approach, with an acceptable postoperative complications rate [36, 37]. On the other hand, Nixon and Lang et al. reported an increasing risk in terms of neck hematoma and generally of postoperative complications as the price for a more radical surgery in elderly patient [38, 39]. Recently, in a meta-analysis Zhao et al. [40] reported a decreased central lymph nodes recurrence rate at most of 1% in patients undergoing pCND, unfortunately associated with increasing postoperative complication rate (mainly hypoparathyroidism and recurrent laryngeal nerve injury). However, Zhao reported also a higher incidence of complications following reoperation for neck recurrence, when compared to complications associated with first operation, especially for permanent hypoparathyroidism (8–27%) [41, 42]. Opposite conclusions were reached by Kim et al. that reported a significantly better prognosis in patients over 65 years old, suggesting a tailored treatment approach especially in case of microcarcinomas [14].

The present series showed a lymph nodes recurrence rate of 17 out of 184 cases in the patient underwent TT alone (9.2%, 10 central recurrence and 7 ipsilateral recurrence) and of 16 out of 187 cases in the patient underwent TT with pCND (8.5%, 8 central recurrence and 8 ipsilateral recurrence), with a difference not statistically significant. There was a difference statically significant between group A and group B in terms of neck hematoma (0.5% vs 3.7%), temporary hypoparathyroidism (11.4% vs 21.4%) and temporary unilateral nerve injury (1.5% vs 6.4%). According to Literature data, the recurrence rate in central lymph nodes compartment in patients aged over 65 years was higher compared to series of younger patients and was associated with a higher tumor staging and an increased rate of tumor multifocality [5, 43, 44]. Considering the patient stratified by age, the Authors reported a double rate of central lymph nodes compartment recurrence in over 75 years old patients, without statistically significant difference with patients aged 65–74 years. Moreover, in the present series a higher incidence of multifocality, of tumor stage and of postoperative complications such as recurrent nerve injury and temporary and definitive hypoparathyroidism, was reported in patients over 75 year old. These results are consistent with current Literature describing more aggressive pattern for DTC in very old and very young patients [16]. The improved staging by the discovery of unexpected lymphnodes metastases (21.9%) was the main advantage in the pCND group, especially in case of microcarcinomas, even if can cause upstaging and potential overtreatment with the risk of iatrogenic morbidity [5]. Conzo et al., in a large series on DTC patients, reported a similar incidence in TT patients and TT associated with pCND, followed by RAI administration and TSH suppression, in terms of local recurrence [5]. Moreover, in case of relapse, in their experience reoperations was not associated with increased postoperative complications, if performed in referral center by experienced endocrine surgeons [45–50].

The study has some limitations. First, a unique definition of “old” patients is not available in Literature and various Authors adopt different age cutoff, eg 60,65,70,75 years, to classify elderly patients. The Authors considered age > 75 years old as a major risk factor for increased recurrence rate and aggressive tumor behavior. Second, the retrospective manner of the study did not allow any correction of confounding factors. Third, given the indolent clinical course of DTC, this study was conducted with a relatively short follow up period (53% completed 5 years follow up), limiting the analysis on late recurrence and death.

## Conclusion

The role of pCND in DTC is still controversial, especially in elderly patients. Aging lead to a decreased quality of life due to the frequent comorbidities, which could worsen in case of postoperative complications. An aggressive approach, with extensive prophylactic lymph node dissection, should be carefully evaluated. The present series showed a similar low recurrence rate between TT and TT associated with pCND, with an increased postoperative complications in the lymphadenectomy group and in patients over 75 years old, advocating a tailored surgical approach in elderly population. Further randomized controlled study are needed in order to evaluate the clear advantages of pCND, especially in elderly patients.

## Abbreviations

CI: Confidential interval
DTC: Differentiated Thyroid Cancer
FNC: Fine needle aspiration cytology
OR: Odds ratio
pCND: Prophylactic central neck dissection
PTC: Papillary thyroid cancer
TT: Total thyroidectomy
US: Ultrasonography

## Authors’ information

Gambardella Claudio and Mauriello Claudio are PhD student in Medical, Clinical and Sperimental Sciences at Univerity of Campania “Luigi Vanvitelli” – Naples, Italy.
